# Changes in Cardiovascular Health Status and the Risk of New-Onset Hypertension in Kailuan Cohort Study

**DOI:** 10.1371/journal.pone.0158869

**Published:** 2016-07-19

**Authors:** Fei Gao, Xiaoxue Liu, Xizhu Wang, Shouhua Chen, Jihong Shi, Ying Zhang, Shouling Wu, Jun Cai

**Affiliations:** 1 Department of Opthalmology, LKS Faculty of Medicine, The University of Hong Kong, Hong Kong, China; 2 Department of Cardiology, Tangshan People's Hospital, North China University of Science and Technology, Tangshan, China; 3 Department of Health Care Center, Kailuan Hospital, North China University of Science and Technology, Tangshan, China; 4 Department of Cardiology, Kailuan Hospital, North China University of Science and Technology, Tangshan, China; 5 Department of Ultrasonography, Hospital Affiliated to North China University of Science and Technology, Tangshan, China; 6 Department of Hypertension, State Key Laboratory of Cardiovascular Disease, National Center for Cardiovascular Diseases, Fuwai Hospital, Chinese Academy of Medical Sciences and Peking Union Medical College, Beijing, China; Scuola Superiore Sant'Anna, ITALY

## Abstract

American Heart Association cardiovascular health metrics are intimately related to cardiovascular diseases. Acting as a key independent risk factor for high morbidity and mortality of cardiovascular diseases, hypertension and its relationship between health status get urgent attention. While the influence of individual health status changes and the future risk of new-onset hypertension is rarely understood, the present study applied this construct to assess the changes of cardiovascular health status and the morbidity of hypertension in Kailuan cohort study in north China. The Cardiovascular Health Score (CHS) was evaluated by the follow-ups of 2006–2007, 2008–2009, 2010–2011 and 2012–2013. The study population (n = 19381) was divided into 5 groups based on the changes in their CHS score between the first two follow-ups (△CHS) of 2006–2007 and 2008–2009 (≤-2, -1, 0, 1, ≥2). The morbidity of hypertension was collected during 2010–2011 and 2012–2013 follow-ups. Data analysis showed that during a median follow-up of 3.79±0.96 years, morbidity of hypertension had a graded relationship with △CHS. As △CHS scored from low to high, the standardized morbidity of hypertension for all participants were 81.40, 75.47, 68.37, 71.43 and 83.13 per 1000 person-year, respectively. An increased △CHS score of 1 was associated with a 10% decrease in the future risk of new-onset hypertension(HR: 0.90, 95% CI: 0.88–0.92). In conclusion, there was a strong inverse relationship between the incidence of new-onset hypertension and elevation of cardiovascular health metrics. Population-wide prevention, especially the promotion of lifestyle improvements, is critical to reducing the morbidity of new-onset hypertension.

## Introduction

To evaluate cardiovascular health level and decrease the morbidity of cardiovascular diseases, the American Heart Association (AHA) defined seven behaviors and risk factors to serve as health metrics [1], which include: smoking status, body mass index, physical activity, healthy dietary score, total cholesterol, blood pressure, and fasting blood glucose. With those risk factors, the AHA subsequently described three stages for each metric to reflect poor, intermediate, and ideal cardiovascular health status [1]. Multiple studies in different countries and ethnic backgrounds revealed that ideal cardiovascular health metrics can significantly decrease the incidence of coronary heart disease [2–5], stroke [6,7], atherosclerosis [8], vascular intima-media thickness [9], as well as cancer [10]. Furthermore, ideal cardiovascular health metrics also have a distinct protective effect on risk factors or subclinical symptoms of cardiovascular diseases, including high-sensitivity C-reactive protein (hs-CRP) [11], hypercholesterolemia [12], uric acid [13,14], carotid intima-media thickness [9,15],asymptomatic intracranial artery stenosis [16], and pulse wave velocity [17,18].

Hypertension is an internationally-accepted risk factor for the development of cardiovascular diseases, which can be detected before manifesting many subclinical symptoms. A prospective study performed by Dolovich *et al*. has demonstrated that the cardiovascular health awareness program (CHAP) is associated with a reduction in systolic and diastolic blood pressure [19]. Our recent study also proves that ideal cardiovascular health behaviors and factors play a prevent role on the development of hypertension [20]. However, the knowledge about the impact and association of the dynamic changes of health metrics on the future development of new-onset hypertension are still limited currently. Therefore, we analyzed data from the Kailuan study (Unique identifier: ChiCTRTNC-11001489) to investigate the relationship between the fluctuation of the modified AHA metrics and the morbidity of hypertension in the northern Chinese cohort located in the industrial city of Tangshan.

## Materials and Methods

### Study Design and Participants

The Kailuan study has been described previously [5,21].Briefly, it is a prospective cohort study based on the Kailuan community in Tangshan,an industrial city located in the north of China. From June 2006 to October 2007, baseline health records were collected in eleven local hospitals responsible for healthcare of the community. Residents who met the following criteria were recruited into the Kailuan study: (1) age≥18 years; (2) provided with informed consent; (3) updated their health status according to the follow-up protocol every 2 years. All participants underwent questionnaire assessment, clinical examination, and laboratory assessments. Specially trained doctors and nurses used standard protocols for all measurements and evaluations [5,22]. The follow-up visits were documented during the years of 2008–2009, 2010–2011, 2012–2013. In our study, participants were excluded if they had any of the following characteristics during the 2006–2007 and 2008–2009 assessment: incomplete data was available, the patient had a history of hypertension, or the patient demonstrated a systolic pressure≥140mmHg or diastolic pressure≥90mmHg. Follow-up evaluations included biannual measurement of laboratory parameters and recording of adverse events. The study was approved by the Ethics Committees of Kailuan General Hospital, following the guidelines outlined by the Helsinki Declaration. All participants agreed to participate in the study and provided written informed consent.

### Questionnaire Assessment, Blood Pressure Measurement and Laboratory Assessments

Questionnaires were administered by research doctors in person. Information including demographic and socioeconomic data, education level, the average income of each family member, medical history, alcohol consumption, smoking status, dietary data, physical exercise were obtained [21]. Smoking status was self-reported as “never”, “former”, or “current". Physical exercise was self-evaluated by the type and frequency and was classified as “very active”, “moderately active” or “inactive”. For dietary data, as the Kailuan study began in 2006, it did not include an assessment of vegetable intake initially. In consideration of the relationship between high salt intake and the morbidity of cardiovascular diseases, an assessment of salt intake was added to the routine questionnaire. Based on responses to questions related to salt preference, salt intake was classified as “low”, “medium” or “high”. We used “low” salt intake as a surrogate of ideal diet in this study for salted food intake is a serious issue in northern China. The questionnaires provided an approximation of whether an individual’s diet was ‘‘ideal,” ‘‘intermediate,” or ‘‘poor”. The average monthly income of each family member was reported as “≤¥600” or “≤¥600”. Education level was categorized as “none or primary school”, “middle school” or “college or university”. Alcohol intake was defined as drinking alcohol more than 100 ml per day at least for 1 year. Blood pressure (BP), rest heart rate (RHR), uric acid (UA), fasting blood glucose(FBG), total cholesterol and triglycerides (TC) were measured with standard procedure as previously described [16,21].

### Cardiovascular Health Scores

Participants who never smoked were considered as ideal health while former smokers were considered as intermediate health. Participants who were currently smoking were defined as the evidence of poor health. Participants with a BMI of <25, 25–30, and ≥30 kg/m^2^ had ideal, intermediate, and poor health, respectively. For physical exercise, ideal, intermediate and poor health were defined as ≥80 minutes, 1–79 minutes, and 0 minutes of moderate or vigorous exercise per week, respectively. As a surrogate of ideal diet, low salt intake was considered as the ideal health, while participants with intermediate salt intake were assigned to intermediate health and those with high salt intake were classified as poor health. Ideal, intermediate, and poor health for TC were defined as <200 mg/dL, 200–239 mg/dL, and ≥240 mg/dL, respectively, if they were not receiving treatment. Participants taking lipid-lowering agents were identified as intermediate health if they had ideal TC level and as poor health if they had the intermediate level. In this study, for participants with hypertension at the baseline were excluded, there had two states of BP level at the baseline: SBP <120 mmHg and DBP <80 mmHg were defined as ideal, SBP of 120–139 mmHg or DBP of 80–89 mmHg were defined as intermediate. Ideal health, intermediate health, and poor health were defined as fasting plasma glucose<100 mg/dL, 100–125 mg/dL, and≥126 mg/dL, respectively, for participants having no treatment of hyperglycemia. If they were receiving hypoglycemic agents, participants were classified as intermediate if they had an ideal FBG level and as poor if they had intermediate FBG level.

### △CHS and New-Onset Hypertension Events

To capture individual-level changes in cardiovascular health factors and behaviors, all participants were grouped based on the composite, individual-level CHS system created by Mark D. Huffman *et al*. [23]. This system is based on the individual-level composite score of all 7 cardiovascular health behaviors and health factors (poor = 0 points; intermediate = 1 point; ideal = 2 points; total scale: 0–14 points). The participants were divided into 5 subgroups according to their changes in CHS during 2006–2007 and 2008–2009 (CHS got in the later examination minus CHS got in the former examination, △CHS), which were ≤-2, -1, 0, 1, ≥2, respectively.

All participants received a baseline examination from 2006 and received follow-up evaluations through 2013 or to the date of death or loss to follow-up. New-onset hypertension was considered as the endpoint event, which was ascertained by surveying each year’s discharge lists from local hospitals and by contacting participants annually for a history of new-onset hypertension events in the Kailuan study. For patients diagnosed with hypertension, professional doctors made the diagnosis and evaluation independently according to our new-onset hypertension diagnostic criteria, which were defined as follows: participants with SBP of less than 140mmHg, DBP less than 90mmHg, no history of hypertension and no use of antihypertensive drugs at the first two examinations of the study (2006–2007 and 2008–2009), had their SBP of at least 140mmHg, DBP of at least 90mmHg and/or used of antihypertensive drugs during the last two follow-up periods.

### Statistical Analyses

Statistical analyzes were performed using SAS software, version 9.3 (SAS Institute, Cary, North Carolina, USA). Data were presented as means ± SD for continuous variables and as frequencies and percentages for categorical variables. We used the Student’s t-test or Analysis of variance (ANOVA) test for the comparison of normally distributed parameters and the Wilcoxon test for the comparison of the non-parametric variable. The Chi-squared test was applied for the comparison of a categorical variable. Cox proportional hazard regression analysis was performed to evaluate the relationship between △CHS and new-onset hypertension. We also estimated the association between CHS metrics and new-onset hypertension stratified by sex and age groups. All statistical tests were 2-sided with the significant level set at 0.05.

## Results

**Fig 1:**
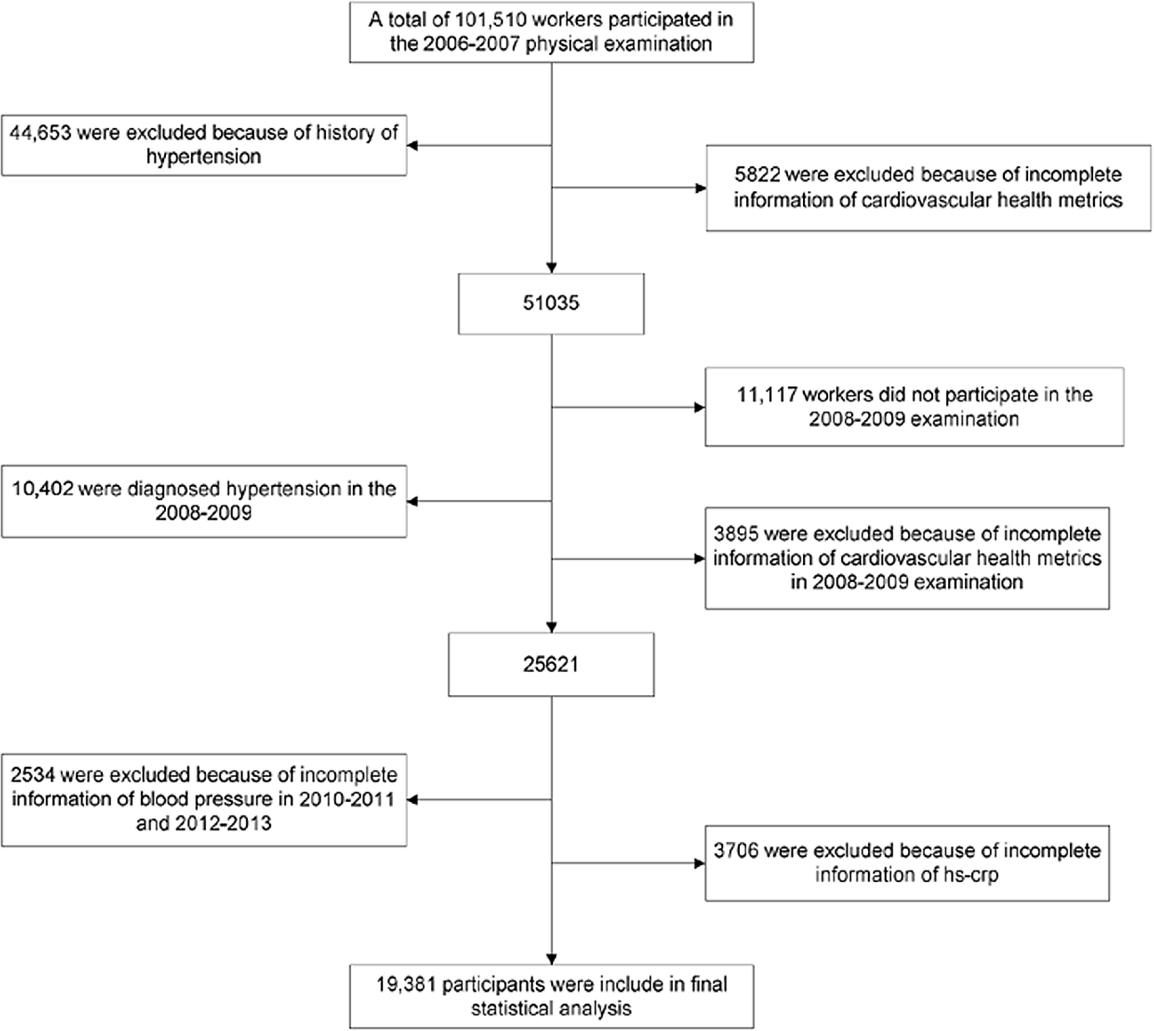
Selection of Kailuan study participants for analysis.

We analyzed data from 19,381 participants which included 14,022 men (45.38 ± 11.77 years) and 5,359 women (43.85 ± 10.13 years) (Fig 1). The baseline characteristics of total participants in five groups according to △CHS were showed (Table 1). Age, sex, baseline cardiovascular health scores, hs-CRP, UA, RHR, smoking status and alcohol intake were analyzed. All of the factors in each group showed significant statistical differences (P<0.05). Clinical characteristics of participants during 2006–2007 were grouped according to whether or not participated the 2008 survey (Table 2). Participants who did not attend 2008–2009 follow-up were significantly older and demonstrated higher levels of hs-CRP, UA and RHR. Notably, the proportion of non-participants was especially high among males, which would be predicted to have lower CHS.

**Table 1:** Basic Characteristics in 2006 according to the change of cardiovascular health scores (△CHS) from 2006 to 2008.

The change of Cardiovascular Health Scores						
	Quintile one (△CHS≤-2)	Quintile two (△CHS = -1)	Quintile three (△CHS = 0)	Quintile four (△CHS = 1)	Quintile five (△CHS≥2)	P
No. of included participants	3863(19.93)	4011(20.70)	5044(26.03)	3671(18.94)	2792(14.41)	
The change of cardiovascular health scores(△CHS)	-2(-9,-2)	-1(-1,-1)	0(0,0)	1(1,1)	2(2,8)	<0.0001
Age, years	44.91±11.24	44.21±11.27	44.54±11.52	45.25±11.32	46.53±11.30	<0.0001
Men, n (%)	3199(82.81)	2926(72.95)	3358(66.57)	2455(66.88)	2084(74.64)	<0.0001
Cardiovascular Health scores in 2006	10.48±1.42	10.15±1.50	9.90±1.56	9.40±1.58	8.30±1.68	<0.0001
High sensitive C-reactive protein, mg/L	1.74±4.03	1.72±4.58	1.84±5.07	1.79±4.63	1.79±4.99	<0.0001
Education, n (%)						
Elementary school	176(4.56)	166(4.14)	213(4.22)	224(6.11)	215(7.71)	<0.0001
High school	2808(72.71)	2723(67.92)	3449(68.41)	2411(65.73)	1884(67.53)	
Collage or above	878(22.73)	1120(27.94)	1380(27.37)	1033(28.16)	691(24.77)	
Income>600 RMB/month n (%)	223(5.78)	293(7.31)	386(7.66)	246(6.71)	179(6.42)	<0.0001
Alcohol drinking, n (%)						
Never	2450(63.42)	2348(58.57)	3053(60.58)	2115(57.68)	1392(49.87)	<0.0001
Past	92(2.38)	102(2.54)	122(2.42)	80(2.18)	93(3.33)	
Current, <1 times/d	821(21.25)	980(24.44)	1208(23.97)	906(24.71)	713(25.55)	
Current, 1+times/d	500(12.9)	579(14.44)	657(13.04)	566(15.43)	593(21.25)	
2006 Uric acid	276.44±75.73	273.87±75.26	269.12±76.30	272.47±78.76	277.25±79.33	<0.0001
2006 Resting heart rate	72.05±9.33	72.39±9.23	72.50±9.13	72.41±9.17	72.77±9.51	<0.0001
2008 High sensitive C-reactive protein, mg/L	2.47±4.36	2.53±5.06	2.59±6.43	2.62±5.52	2.66±5.16	<0.0001
2008 Uric acid	273.60±78.89	270.61±78.33	266.25±76.44	270.73±78.48	273.40±78.10	<0.0001

**Table 2:** Clinical Characteristics in 2006 according to the participants who participated or not participated the 2008 survey.

	participants who participate the 2008 survey	participants who did not participate the 2008 survey	P difference
No. of participants	29515(72.64)	11117(27.36)	
Age, years	45.80±11.86	51.79±13.12	<0.001
Men, n (%)	21057(70.20)	8459(79.51)	<0.001
Cardiovascular Health scores	9.76±1.71	9.60±1.70	<0.001
High sensitive C-reactive protein, mg/L	1.87±4.87	2.31±6.69	<0.001
Uric acid	277.43±77.96	283.37±77.75	<0.001
Resting heart rate	72.40±9.28	73.17±10.38	<0.001

### Incidence of New-Onset Hypertension

During a median follow-up of 3.79±0.96 years, as △CHS scored from -2 to 2, the standardized morbidity rate of hypertension for all participants were 81.40, 75.47, 68.37, 71.43 and 83.13 per 1000 person-year, respectively (Table 3). In order to adjust the confounding factors, the analyzes were performed by adjusting for age, sex and baseline cardiovascular health metrics (model 1) and further adjusting for education, income and alcohol use (model 2). Then, the models were further adjusted for hs-CRP, UA, RHR got in 2006 (model 3) and hs-CRP, UA, RHR measured by the 2008 follow-up (model 4). In model 4 adjusting for all of the confounding factors above, compared with the first quintile (lowest △CHS), the HR value (95%CI) of the quintiles two, three, four, and five were 0.91(0.84–0.99), 0.79(0.73–0.86), 0.73(0.67–0.80) and 0.68(0.62–0.74) respectively, demonstrating that the incidence of hypertension decreased as △CHS increased. In model 4, we also got the result that the morbidity of hypertension decreased by approximately 10% as △CHS increased by a score of 1 (HR: 0.9095% CI: 0.88–0.92).

**Table 3:** Hazard ratios (HRs) and 95% confident intervals (95% CIs) of hypertension according to change of cardiovascular health scores (△CHS) from 2006 to 2008.

	Quintile one (△CHS≤-2)	Quintile two (△CHS = -1)	Quintile three (△CHS = 0)	Quintile four (△CHS = 1)	Quintile five (△CHS≥2)	One score increase (△CHS)	P for trend
Case number	1208	1169	1342	1020	896	5635	
Incidence rate, per 1,000 person-year	81.40	75.47	68.37	71.43	83.13	75.12	
Model 1 *	1	0.90(0.83–0.98)	0.78(0.72–0.84)	0.72(0.66–0.79)	0.66(0.60–0.73)	0.90(0.88–0.92)	<0.001
Model 2 †	1	0.91(0.84–0.98)	0.78(0.72–0.84)	0.73(0.66–0.79)	0.66(0.60–0.73)	0.90(0.88–0.92)	<0.001
Model 3 ‡	1	0.91(0.84–0.98)	0.78(0.72–0.85)	0.73(0.67–0.80)	0.67(0.61–0.74)	0.90(0.88–0.92)	<0.001
Model 4 #	1	0.91(0.84–0.99)	0.79(0.73–0.86)	0.73(0.67–0.80)	0.68(0.62–0.74)	0.90(0.88–0.92)	<0.001

* Adjusted for age (years), sex and cardiovascular health scores in 2006.

†Adjusted for as model 1 plus education level (elementary school, high school or college or above), income level (income>600 RMB/month or income≤600 RMB/month) and drinking (never, past, current, <1times/d or current, 1+times/d).

‡ Adjusted for as model 2 plus High sensitive C-reactive protein, UA and RHR in 2006.

# Adjusted for as model 3 plus High sensitive C-reactive protein, UA and RHR in 2008.

### Risk of Hypertension According to Age, Sex, Baseline CHS Scores and hs-CRP Level

We showed the detailed information about the morbidity of hypertension in five △CHS subgroups when further analyzed according to sex, age, CHS scores in 2006 and hs-CRP level in 2006 (Table 4). After adjustment for age, sex, baseline cardiovascular health scores, education, income, alcohol intake and RHR, the risk of new-onset hypertension in the fifth quintile (△CHS≥2) decreased by 51% for females (HR: 0.49, 95% CI: 0.39–0.62) and by 25% for males (HR: 0.75, 95% CI: 0.67–0.83) when compared with the first quintile. In the participants aged ≤60 years and those>60 years, the risk of hypertension were reduced by 31% (HR: 0.69, 95% CI: 0.63–0.77) and 37% (HR: 0.63, 95% CI: 0.48–0.82), respectively. The risk of hypertension was decreased by 16% (HR: 0.84, 95% CI: 0.62–1.12) and 34% (HR: 0.66, 95% CI: 0.59–0.73) when determined for participants whose baseline cardiovascular health scores were ≤ 7 or>7, respectively. In the groups with hs-CRP ≤3mg/L and>3mg/L, the risk of hypertension was decreased by 31% (HR: 0.69, 95% CI: 0.62–0.76) and 34% (HR: 0.66, 95% CI: 0.51–0.85), respectively. In summary, when analyzed by age, sex, baseline CHS, or hs-CRP level, the incidence of hypertension showed decline trend as the △CHS increased from -2 to 2. With increasing △CHS, the risk of new-onset hypertensive events in the future was significantly diminished (*P*<0.01).

**Table 4:** Incidence of hypertension in different groups according to the △CHS.

	Quintile one (△CHS ≤-2)	Quintile two (△CHS = -1)	Quintile three (△CHS = 0)	Quintile four (△CHS = 1)	Quintile five (△CHS ≥2)	One score increase (△CHS)	P for trend	P-interaction^&^
Sex								0.61
Women, Case number	172(25.90)	247(22.76)	305(18.09)	236(19.41)	163(23.02)	1123(20.96)		
Incidence rate, per 1,000 person-year	66.12	57.36	45.15	48.59	57.57	52.60		
Hazard ratio ‡	1	0.90(0.74–1.09)	0.70(0.58–0.84)	0.63(0.51–0.77)	0.49(0.39–0.62)	0.84(0.80–0.88)	<0.001	
Men, Case number	1036(32.39)	922(31.51)	1037(30.88)	784(31.93)	733(35.17)	4512(32.18)		
Incidence rate, per 1,000 person-year	84.64	82.44	80.56	83.20	92.23	84.08		
Hazard ratio ‡	1	0.92(0.84–1.01)	0.84(0.77–0.92)	0.78(0.71–0.86)	0.75(0.67–0.83)	0.93(0.90–0.95)	<0.001	
Age years								0.65
≤60ys,Case number	1065(29.83)	1028(27.73)	1159(25.13)	870(26.10)	763(30.59)	4885(27.57)		
Incidence rate, per 1,000 person-year	77.20	71.33	64.19	66.54	78.72	70.76		
Hazard ratio ‡	1	0.91(0.83–0.99)	0.80(0.73–0.87)	0.73(0.66–0.80)	0.69(0.63–0.77)	0.91(0.89–0.93)	<0.001	
>60ys,Case number	143(48.81)	141(46.38)	183(42.36)	150(44.38)	133(44.63)	750(45.05)		
Incidence rate, per 1,000 person-year	136.89	130.84	116.41	124.50	122.50	125.32		
Hazard ratio ‡	1	0.94(0.75–1.19)	0.78(0.63–0.98)	0.77(0.61–0.98)	0.63(0.48–0.82)	0.90(0.84–0.95)	<0.001	
Cardiovascular Health scores in 2006								0.64
≤7, Case number	52(48.15)	84(46.41)	153(41.46)	199(42.07)	361(41.73)	849(42.54)		
Incidence rate, per 1,000 person-year	129.41	127.47	113.02	114.21	112.43	115.23		
Hazard ratio ‡	1	1.04(0.74–1.48)	0.92(0.67–1.26)	0.90(0.66–1.22)	0.84(0.62–1.12)	0.95(0.90–1.00)	0.036	
>7, Case number	1156(30.79)	1085(28.33)	1189(25.43)	821(25.67)	535(27.76)	4786(27.53)		
Incidence rate, per 1,000 person-year	80.06	73.16	65.07	65.48	70.69	70.75		
Hazard ratio ‡	1	0.90(0.83–0.98)	0.78(0.72–0.84)	0.72(0.65–0.78)	0.66(0.59–0.73)	0.90(0.88–0.92)	0.001	
High sensitive C-reactive protein in 2006								0.79
≤3mg/L, Case number	1018(30.58)	994(28.65)	1123(25.75)	858(27.39)	770(31.82)	4763(28.50)		
Incidence rate, per 1,000 person-year	79.32	73.70	65.90	70.31	82.19	73.35		
Hazard ratio‡	1	0.91(0.83–0.99)	0.79(0.72–0.86)	0.75(0.68–0.82)	0.69(0.62–0.76)	0.91(0.89–0.93)	<0.001	
>3mg/L, Case number	190(35.58)	175(32.35)	219(32.11)	162(30.06)	126(33.87)	872(32.68)		
Incidence rate, per 1,000 person-year	94.68	87.40	84.72	78.03	89.36	86.51		
Hazard ratio ‡	1	0.95(0.77–1.17)	0.84(0.69–1.03)	0.70(0.56–0.87)	0.66(0.51–0.85)	0.89(0.84–0.94)	<0.001	

‡ Adjusted for age (years), sex, cardiovascular Health scores in2006, education level (elementary school, high school or college or above), income level (income>600 RMB/month or income≤600 RMB/month), drinking (never, past, current, <1times/d or current, 1+times/d), high sensitive High sensitive C-reactive protein (hs-CRP), uric acid (UA) and resting heart rate (RHR) in 2006 and 2008.

### Sensitivity Analysis

To explore whether each change of the cardiovascular health metrics accounted for the relationship between △CHS and the incidence of hypertension, we eliminated one of the factors and evaluated △CHS of the rest six factors (Table 5). The result illustrated that, after removing each factors (smoking, salt intake, physical exercise, total cholesterol, BP, blood glucose and BMI), the total HR (95%CI) for one score increase of △CHS was 0.91(0.90–0.93), 0.92(0.91–0.94), 0.92(0.91–0.94), 0.93(0.91–0.95), 0.93(0.92–0.95), 0.92(0.91–0.94) and 0.92(0.91–0.94), respectively. It should be noticed that this tendency was found both in male and female. Data from this analysis indicated that the residual △CHS remain a statistically significant inverse correlation with hypertension after removing any one of the 7 indexes, regardless of the gender.

**Table 5:** Hazard ratios (HRs) and 95% confident intervals (95% Cis) of hypertension according to change of cardiovascular health scores (△CHS) from 2006 to 2008, after one individual cardiovascular health is removed from the total score.

Removed component	total		women		men	
	HR (95%CI) for one score increase	P for trend	HR (95%CI) for one score increase	P for trend	HR (95%CI) for one score increase	P for trend
Smoke‡	0.91(0.90–0.93)	<0.001	0.86(0.82–0.90)	<0.001	0.92(0.91–0.94)	<0.001
Salt‡	0.92(0.91–0.94)	<0.001	0.86(0.82–0.90)	<0.001	0.94(0.92–0.96)	<0.001
Physical exercise‡	0.92(0.91–0.94)	<0.001	0.81(0.78–0.85)	<0.001	0.95(0.93–0.97)	<0.001
Total cholesterol‡	0.93(0.91–0.95)	<0.001	0.87(0.84–0.92)	<0.001	0.95(0.93–0.97)	<0.001
Blood pressure‡	0.93(0.92–0.95)	<0.001	0.88(0.84–0.92)	<0.001	0.95(0.93–0.97)	<0.001
Fasting blood glucose‡	0.92(0.91–0.94)	<0.001	0.87(0.83–0.91)	<0.001	0.94(0.92–0.96)	<0.001
Body mass index‡	0.92(0.91–0.94)	<0.001	0.86(0.82–0.90)	<0.001	0.94(0.93–0.96)	<0.001

‡ Adjusted for age (years), sex, cardiovascular health scores in 2006, education level (elementary school, high school or college or above), income level (income>600 RMB/month or income≤600 RMB/month), drinking (never, past, current, <1times/d or current, 1+times/d),High sensitive C-reactive protein (hs-CRP), uric acid (UA) and resting heart rate (RHR) in 2006 and 2008.

### The Influence of △CHS to hs-CRP, UA and RHR

To learn whether △CHS is associated with the change of hs-CRP, UA or RHR levels during the follow-up period, multivariate linear regression analysis was performed (Table 6). After adjusting for age, sex, baseline cardiovascular health metrics, education and income level, the results demonstrated that △CHS had an inverse relationship with hs-CRP level, as it decreased by 0.088mg/L when △CHS increased by 1 score (*P*<0.001). However, no significant differences were found between the relationship of △CHS and UA and RHR.

**Table 6:** Mean difference (95% confidence interval) in High sensitive C-reactive protein, UA, HR increase during 2008–2010, according to change of cardiovascular health score (△CHS) from 2006 to 2008.

		Quintile one (△CHS≤-2)	Quintile two (△CHS S = -1)	Quintile three (△CHS = 0)	Quintile four (△CHS = 1)	Quintile five (△CHS≥2)	One score increase (△CHS)	P for trend
Hs-CRP	Model 1 *	0(ref)	-0.2653	-0.2748	-0.3192	-0.4074	-0.0883	0.03
	Model 2 †	0(ref)	-0.2734	-0.2850	-0.3272	-0.4031	-0.0880	0.03
UA	Model 1 *	0(ref)	2.0713	0.0149	-0.3643	-2.5784	-0.6921	0.10
	Model 2 †	0(ref)	2.0340	1.5403	1.6955	1.9308	-0.6822	0.11
RHR	Model 1 *	0(ref)	-0.2742	-0.0358	0.1581	-0.1881	0.0174	0.78
	Model 2 †	0(ref)	-0.2825	-0.0399	0.1621	-0.2036	0.0160	0.79

* Adjusted for age (years), sex, cardiovascular health scores in 2006,High sensitive C-reactive protein(CRP), uric acid (UA) and resting heart rate(HR) in 2006.

†Adjusted for as model 1 plus education level (elementary school, high school or college or above), income level (income>600 RMB/month or income≤600 RMB/month) and drinking (never, past, current, <1times/d or current, 1+times/d).

## Discussion

In this cohort study analysing data from community CHS conducted in the north city of China for years, we found that there was a strong inverse relationship between the incidence of new-onset hypertension and △CHS. The CHS score changes can act as an independent predictive factor for the future development of hypertension.

The AHA defined a set of ideal cardiovascular health metrics that aimed to measure the improvement in overall cardiovascular health in all Americans toward 2020 Impact Goal [1]. Acting as the reliable indicators of cardiovascular protection functions[24],an increased number of ideal cardiovascular health metrics at baseline is associated with substantially declined cardiovascular event rates and mortality in short and long-term follow-up [2,6,9,12,25,26]. However, these cross-sectional studies only performed their analysis with a single time point cardiovascular health status and ignored the effect of the dynamic changes of the status to the morbidity or mortality associated with cardiovascular diseases. To our knowledge, our study is the first large-scale prospective cohort study to evaluate the longitudinal effects of alterations in the CHS metrics on the incidence of new-onset hypertension. As our previous study showed that only 0.1% participants met all seven ideal cardiovascular health behaviors and factors, and only 9.1% met ≥5 ideal health metrics in the north of China [5], whether the adult individuals with low health status at baseline could decrease their future risk of cardiovascular diseases became a pressing question. Therefore, our study about the relationship between △CHS and the risk of hypertension has practical implications for the cardiovascular health of the adult population in China.

Being exhaustively studied, hypertension has been found highly prevalent in older age and male[27–32].Studies in recent years have also proved that people with a better baseline level of CHS have a strong and inverse association with risk of cardiovascular diseases and blood pressure level[3,20,33]. In our study, the change of cardiovascular health scores is significantly associated with the morbidity of new-onset hypertension. The increasing of △CHS decreased the incidence of hypertension dramatically in the 4-year follow-up, the association, however, was still held after adjustment for age, sex, baseline cardiovascular metrics, education, income level, alcohol intake, baseline hs-CRP, UA and RHR. Based on these results, we can predict that adult population can reduce their risk of hypertension in the future as they promote their cardiovascular health scores, regardless of the basic health status they had in the past. We should especially notice that people with different sex, age, and baseline CHS can all get the benefit outcomes at different levels from this improvement. Female, participants aged less than 60 years, and those with baseline CHS>7 may benefit more from the improvement of cardiovascular risk factors and behaviors, which are in accordance with the epidemiology of hypertension.

The sensitivity analysis identified whether any of the 7 ideal cardiovascular metrics may have a greater influence on the incidence of hypertension. The analyzed data showed that the risk of developing hypertension had a significantly and similar decline trend with the increase of △CHS after removing each one of the 7 indexes. Meanwhile, it should be noticed that women have a lower incidence of hypertension than men, which is consistent with our above analysis. Together, these results provide evidence that all of the seven CHS factors are equally important and suggest that raising △CHS may be a viable method by which people can avoid the future development of hypertension while women might benefit more from this improvement.

Previous studies have shown that ideal cardiovascular health metrics have a protective role in the defense against coronary heart disease [2,12], stroke [6], and vascular intima-media thickening [9]. Acting as an inflammation marker and involved in the pathogenesis of atherosclerosis, CRP has been identified as cardiovascular risk factors [34–37]. Studies found a significant association between CRP level and subsequent coronary heart disease mortality in middle-aged men, women, and elderly people [35,38,39]. Our previous study also identified that ideal cardiovascular health metrics had an inverse relationship with serum hs-CRP level, both in general and hypertension population [11,40]. In our current study, hs-CRP significantly decreased as △CHS increased. As hs-CRP is an inflammatory marker and risk factor associated with cardiovascular diseases, this result demonstrates that improvement of CHS can decrease the inflammation status and as a consequence might reduce the incidence of new-onset hypertension.

While offering encouraging results, our study should be considered in light of several limitations. First, the median follow-up time was3.79 years, which may not be long enough to accurately evaluate the endpoint event of new-onset hypertension. Second, for practical reasons, it was not possible to completely consider all of the AHA 2020 health metrics. For example, we used ‘‘low” salt intake as a surrogate of ideal diet, which may result in an underestimation of the effects of diet on morbidity. Third, our sample population had a substantial difference in the distribution of genders, which might be worrisome for selection bias. To mitigate these potential problems, we calculated morbidity rates adjusted for both age and sex, as well as other factors, which proved the similar results in male and female. Fourth, a great number of participants were excluded for lack of follow-up data during 2008 survey, which may also bias the results. However, after comparing participants and non-participants, we found that non-participants were of older age and had higher baseline levels of hs-CRP, UA, and RHR, which are risk factors for hypertension. Therefore, our study may ultimately underestimate the effect of improving cardiovascular health factors on the incidence of hypertension. Lastly, although this study was limited to a population in north China, the participants in the Kailuan study hail from a number of areas and included a multitude careers, and are likely a diverse, representative sample with respect to evaluating cardiovascular risk factors on the development of new-onset hypertension.

In conclusion, although the study had some limitations, our findings demonstrated a strong and independent inverse relationship between changes of CHS and morbidity of hypertension in a Northern Chinese cohort. Although few people have ideal cardiovascular health metrics, improvement of health status still can significantly reduce their future risk of new-onset hypertension and risk factors for cardiovascular diseases. Population-wide prevention, especially the promotion of lifestyle improvements, is critical to promote cardiovascular health in the adult population of China.

## Supporting Information

S1 Checklist STROBE Statement. Completed checklist of items that should be included in reports of observational studies and the relevant text from manuscript.
(DOCX)

S1 Fig Approval notice of Kailuan study. Kailuan General Hosipital Approveal Notice Issued by the Medical Ethics Committee, Approval 5.
(TIF)

## References

[1] Lloyd-Jones DM, Hong Y, Labarthe D, Mozaffarian D, Appel LJ, Van Horn L, et al Defining and setting national goals for cardiovascular health promotion and disease reduction: the American Heart Association's strategic Impact Goal through 2020 and beyond. Circulation. 2010;121(4):586–613. 10.1161/CIRCULATIONAHA.109.192703 . 20089546

[2] Huffman MD, Lloyd-Jones DM, Ning H, Labarthe DR, Guzman Castillo M, O'Flaherty M, et al Quantifying options for reducing coronary heart disease mortality by 2020. Circulation. 2013;127(25):2477–84. 10.1161/CIRCULATIONAHA.112.000769 23661723 PMC3795417

[3] Lachman S, Peters RJ, Lentjes MA, Mulligan AA, Luben RN, Wareham NJ, et al Ideal cardiovascular health and risk of cardiovascular events in the EPIC-Norfolk prospective population study. European journal of preventive cardiology. 2015 10.1177/2047487315602015 . 26336197 PMC6215703

[4] Kim JY, Ko YJ, Rhee CW, Park BJ, Kim DH, Bae JM, et al Cardiovascular health metrics and all-cause and cardiovascular disease mortality among middle-aged men in Korea: the Seoul male cohort study. Journal of preventive medicine and public health = Yebang Uihakhoe chi. 2013;46(6):319–28. 10.3961/jpmph.2013.46.6.319 24349653 PMC3859853

[5] Wu S, Huang Z, Yang X, Zhou Y, Wang A, Chen L, et al Prevalence of ideal cardiovascular health and its relationship with the 4-year cardiovascular events in a northern Chinese industrial city. Circulation Cardiovascular quality and outcomes. 2012;5(4):487–93. 10.1161/CIRCOUTCOMES.111.963694 . 22787064

[6] Dong C, Rundek T, Wright CB, Anwar Z, Elkind MS, Sacco RL. Ideal cardiovascular health predicts lower risks of myocardial infarction, stroke, and vascular death across whites, blacks, and hispanics: the northern Manhattan study. Circulation. 2012;125(24):2975–84. 10.1161/CIRCULATIONAHA.111.081083 22619283 PMC3396556

[7] Zhang Q, Zhou Y, Gao X, Wang C, Zhang S, Wang A, et al Ideal cardiovascular health metrics and the risks of ischemic and intracerebral hemorrhagic stroke. Stroke; a journal of cerebral circulation. 2013;44(9):2451–6. 10.1161/STROKEAHA.113.678839 . 23868276

[8] Ogagarue ER, Lutsey PL, Klein R, Klein BE, Folsom AR. Association of ideal cardiovascular health metrics and retinal microvascular findings: the Atherosclerosis Risk in Communities Study. Journal of the American Heart Association. 2013;2(6):e000430 10.1161/JAHA.113.000430 24252843 PMC3886782

[9] Pahkala K, Hietalampi H, Laitinen TT, Viikari JS, Ronnemaa T, Niinikoski H, et al Ideal cardiovascular health in adolescence: effect of lifestyle intervention and association with vascular intima-media thickness and elasticity (the Special Turku Coronary Risk Factor Intervention Project for Children [STRIP] study). Circulation. 2013;127(21):2088–96. 10.1161/CIRCULATIONAHA.112.000761 . 23613255

[10] Rasmussen-Torvik LJ, Shay CM, Abramson JG, Friedrich CA, Nettleton JA, Prizment AE, et al Ideal cardiovascular health is inversely associated with incident cancer: the Atherosclerosis Risk In Communities study. Circulation. 2013;127(12):1270–5. 10.1161/CIRCULATIONAHA.112.001183 23509058 PMC3685848

[11] Xue H, Wang J, Hou J, Gao J, Chen S, Zhu H, et al Ideal cardiovascular health behaviors and factors and high sensitivity C-reactive protein: the Kailuan cross-sectional study in Chinese. Clinical chemistry and laboratory medicine: CCLM / FESCC. 2014;52(9):1379–86. 10.1515/cclm-2013-0657 . 24791822

[12] Laitinen TT, Pahkala K, Magnussen CG, Viikari JS, Oikonen M, Taittonen L, et al Ideal cardiovascular health in childhood and cardiometabolic outcomes in adulthood: the Cardiovascular Risk in Young Finns Study. Circulation. 2012;125(16):1971–8. 10.1161/CIRCULATIONAHA.111.073585 . 22452832

[13] Qin L, Yang Z, Gu H, Lu S, Shi Q, Xing Y, et al Association between serum uric acid levels and cardiovascular disease in middle-aged and elderly Chinese individuals. BMC cardiovascular disorders. 2014;14:26 10.1186/1471-2261-14-26 24568132 PMC3974065

[14] Du T, Sun X, Lu H, Lin X, Liu Q, Huo R, et al Associations of serum uric acid levels with cardiovascular health factors: differences by sex, age and body mass index in Chinese participants. European journal of internal medicine. 2014;25(4):388–93. 10.1016/j.ejim.2014.03.004 . 24702838

[15] Kulshreshtha A, Goyal A, Veledar E, McClellan W, Judd S, Eufinger SC, et al Association between ideal cardiovascular health and carotid intima-media thickness: a twin study. Journal of the American Heart Association. 2014;3(1):e000282 10.1161/JAHA.113.000282 24385450 PMC3959690

[16] Zhang Q, Zhang S, Wang C, Gao X, Zhou Y, Zhou H, et al Ideal cardiovascular health metrics on the prevalence of asymptomatic intracranial artery stenosis: a cross-sectional study. PloS one. 2013;8(3):e58923 10.1371/journal.pone.0058923 23554958 PMC3595221

[17] Aatola H, Hutri-Kahonen N, Juonala M, Laitinen TT, Pahkala K, Mikkila V, et al Prospective relationship of change in ideal cardiovascular health status and arterial stiffness: the Cardiovascular Risk in Young Finns Study. Journal of the American Heart Association. 2014;3(2):e000532 10.1161/JAHA.113.000532 24614756 PMC4187504

[18] Crichton GE, Elias MF, Robbins MA. Cardiovascular health and arterial stiffness: the Maine-Syracuse Longitudinal Study. Journal of human hypertension. 2014;28(7):444–9. 10.1038/jhh.2013.131 24384629 PMC4079770

[19] Ye C, Foster G, Kaczorowski J, Chambers LW, Angeles R, Marzanek-Lefebvre F, et al The impact of a cardiovascular health awareness program (CHAP) on reducing blood pressure: a prospective cohort study. BMC public health. 2013;13:1230 10.1186/1471-2458-13-1230 24369050 PMC3883556

[20] Gao J, Sun H, Liang X, Gao M, Zhao H, Qi Y, et al Ideal cardiovascular health behaviors and factors prevent the development of hypertension in prehypertensive subjects. Clinical and experimental hypertension. 2015:1–6. 10.3109/10641963.2015.1047938 . 26114351

[21] Liu Y, Chi HJ, Cui LF, Yang XC, Wu YT, Huang Z, et al The ideal cardiovascular health metrics associated inversely with mortality from all causes and from cardiovascular diseases among adults in a Northern Chinese industrial city. PloS one. 2014;9(2):e89161 10.1371/journal.pone.0089161 24586562 PMC3933417

[22] Wang F, Wu S, Song Y, Tang X, Marshall R, Liang M, et al Waist circumference, body mass index and waist to hip ratio for prediction of the metabolic syndrome in Chinese. Nutrition, metabolism, and cardiovascular diseases: NMCD. 2009;19(8):542–7. 10.1016/j.numecd.2008.11.006 . 19188050

[23] Huffman MD, Capewell S, Ning H, Shay CM, Ford ES, Lloyd-Jones DM. Cardiovascular health behavior and health factor changes (1988–2008) and projections to 2020: results from the National Health and Nutrition Examination Surveys. Circulation. 2012;125(21):2595–602. 10.1161/CIRCULATIONAHA.111.070722 22547667 PMC3914399

[24] Maclagan LC, Tu JV. Using the concept of ideal cardiovascular health to measure population health: a review. Current opinion in cardiology. 2015;30(5):518–24. 10.1097/HCO.0000000000000210 . 26196659

[25] Ford ES, Greenlund KJ, Hong Y. Ideal cardiovascular health and mortality from all causes and diseases of the circulatory system among adults in the United States. Circulation. 2012;125(8):987–95. 10.1161/CIRCULATIONAHA.111.049122 . 22291126 PMC4556343

[26] Folsom AR, Yatsuya H, Nettleton JA, Lutsey PL, Cushman M, Rosamond WD, et al Community prevalence of ideal cardiovascular health, by the American Heart Association definition, and relationship with cardiovascular disease incidence. Journal of the American College of Cardiology. 2011;57(16):1690–6. 10.1016/j.jacc.2010.11.041 21492767 PMC3093047

[27] Sandberg K, Ji H. Sex differences in primary hypertension. Biol Sex Differ. 2012;3 Artn 7 10.1186/2042-6410-3-7 . PMC3331829 22417477

[28] Yanes LL, Romero DG, Iliescu R, Reckelhoff JF. A Single Pill to Treat Postmenopausal Hypertension? Not Yet. Curr Top Med Chem. 2011;11(13):1736–41. . 21463249 10.2174/156802611796117667 PMC3462431

[29] Cutler JA, Sorlie PD, Wolz M, Thom T, Fields LE, Roccella EJ. Trends in Hypertension Prevalence, Awareness, Treatment, and Control Rates in United States Adults Between 1988–1994 and 1999–2004. Hypertension. 2008;52(5):818–27. 10.1161/Hypertensionaha.108.113357 . 18852389

[30] Go AS, Mozaffarian D, Roger VL, Benjamin EJ, Berry JD, Blaha MJ, et al Heart Disease and Stroke Statistics-2014 Update A Report From the American Heart Association. Circulation. 2014;129(3):E28–E292. 10.1161/01.cir.0000441139.02102.80 . 24352519 PMC5408159

[31] Liu XF, Tsilimingras D, Paul TK. Prevalence and changes of untreated isolated systolic hypertension among non-Hispanic black adults in the United States. Hypertens Res. 2014;37(7):685–91. 10.1038/hr.2014.58 . 24621464

[32] Still CH, Ferdinand KC, Ogedegbe G, Wright JT. Recognition and Management of Hypertension in Older Persons: Focus on African Americans. J Am Geriatr Soc. 2015;63(10):2130–8. 10.1111/jgs.13672 . 26480975 PMC6310911

[33] Li ZK, Yang X, Wang AX, Qiu J, Wang W, Song QF, et al Association between Ideal Cardiovascular Health Metrics and Depression in Chinese Population: A Cross-sectional Study. Sci Rep-Uk. 2015;5 Artn 11564 10.1038/Srep11564 . PMC4648472 26176196

[34] Lagrand WK, Visser CA, Hermens WT, Niessen HWM, Verheugt FWA, Wolbink GJ, et al C-reactive protein as a cardiovascular risk factor—More than an epiphenomenon? Circulation. 1999;100(1):96–102. . 10393687 10.1161/01.cir.100.1.96

[35] Ridker PM, Hennekens CH, Buring JE, Rifai N. C-reactive protein and other markers of inflammation in the prediction of cardiovascular disease in women. New Engl J Med. 2000;342(12):836–43. 10.1056/Nejm200003233421202 . 10733371

[36] Ridker PM. Clinical application of C-reactive protein for cardiovascular disease detection and prevention. Circulation. 2003;107(3):363–9. 10.1161/01.Cir.0000053730.47739.3c . 12551853

[37] Libby P. Inflammation in atherosclerosis. Nature. 2002;420(6917):868–74. 10.1038/nature01323 . 12490960

[38] Kuller LH, Tracy RP, Shaten J, Meilahn EN. Relation of C-reactive protein and coronary heart disease in the MRFIT nested case-control study. Am J Epidemiol. 1996;144(6):537–47. . 8797513 10.1093/oxfordjournals.aje.a008963

[39] Koenig W, Sund M, Frohlich M, Fischer HG, Lowel H, Doring A, et al C-reactive protein, a sensitive marker of inflammation, predicts future risk of coronary heart disease in initially healthy middle-aged men—Results from the MONICA (Monitoring Trends and Determinants in Cardiovascular Disease) Augsburg Cohort Study, 1984 to 1992. Circulation. 1999;99(2):237–42. . 9892589 10.1161/01.cir.99.2.237

[40] Xue H, Wang JL, Hou JH, Zhu H, Gao JS, Chen SH, et al Association of Ideal Cardiovascular Metrics and Serum High-Sensitivity C-Reactive Protein in Hypertensive Population. PloS one. 2013;8(12). ARTN e81597 10.1371/journal.pone.0081597 . PMC3862853 24349092

