# Supplementary figures and images for: Changes in Cardiovascular Health Status and the Risk of New-Onset Hypertension in Kailuan Cohort Study
Source: PLoS One. 2016 Jul 19;11(7):e0158869. doi: 10.1371/journal.pone.0158869 (PMC4951012; doi:10.1371/journal.pone.0158869)

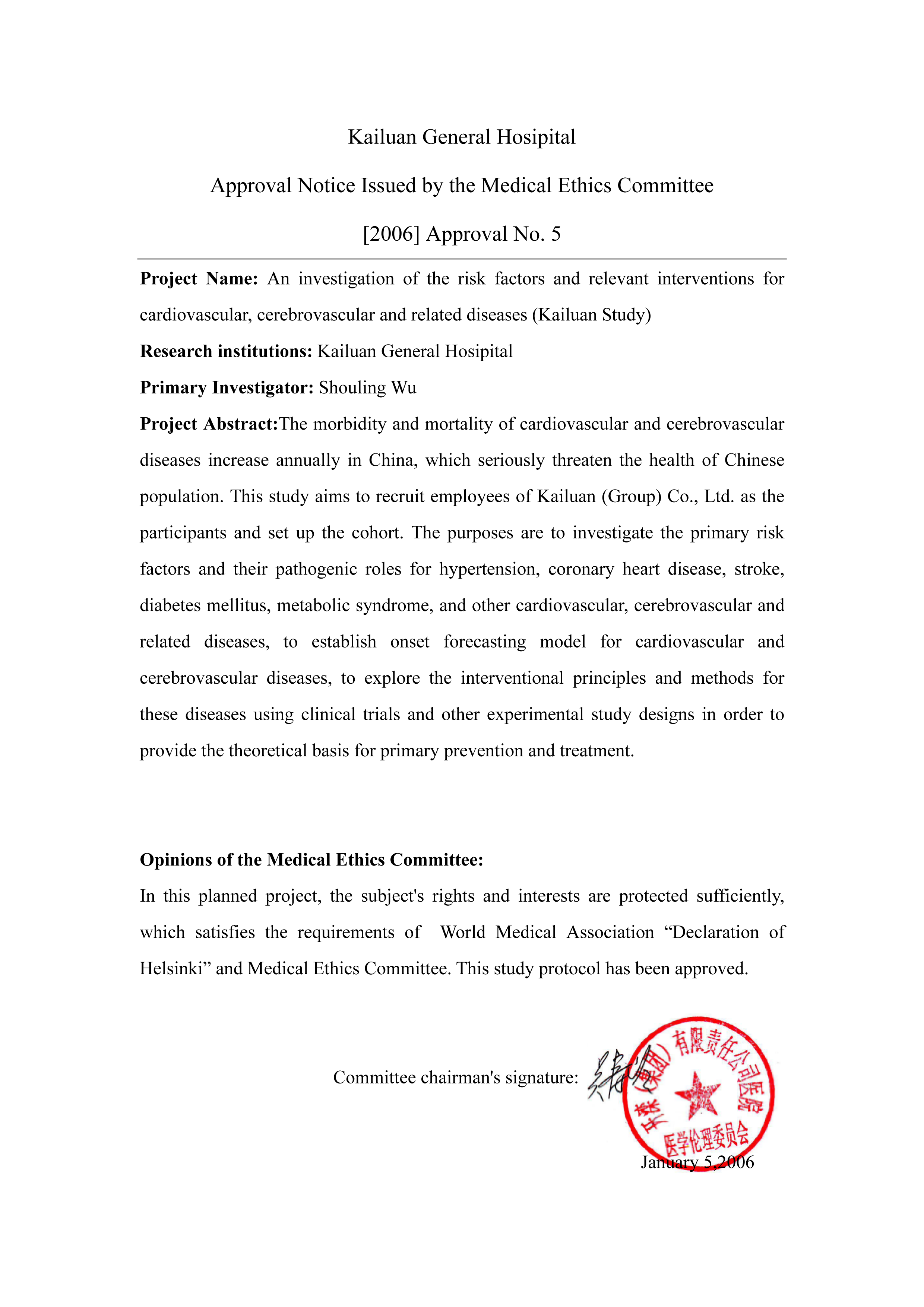

Supplement: S1 Fig — Kailuan General Hosipital Approveal Notice Issued by the Medical Ethics Committee, Approval 5. (TIF) [file pone.0158869.s002.tif]
